# Integrated genomic analyses identify frequent gene fusion events and *VHL* inactivation in gastrointestinal stromal tumors

**DOI:** 10.18632/oncotarget.3731

**Published:** 2015-03-30

**Authors:** Guhyun Kang, Hongseok Yun, Choong-Hyun Sun, Inho Park, Seungmook Lee, Jekeun Kwon, Ingu Do, Min Eui Hong, Michael Van Vrancken, Jeeyun Lee, Joon Oh Park, Jeonghee Cho, Kyoung-Mee Kim, Tae Sung Sohn

**Affiliations:** ^1^ Department of Pathology & Translational Genomics, Samsung Medical Center, Sungkyunkwan University School of Medicine, Seoul, Korea; ^2^ Bioinformatics Laboratory, Samsung SDS Co., Ltd., Seoul, Korea; ^3^ Samsung Cancer Research Institute, Samsung Medical Center, Seoul, Korea; ^4^ Center for Cancer Companion Diagnostics, Samsung Medical Center, Seoul, Korea; ^5^ Department of Medicine, Division of Hematology-Oncology, Samsung Medical Center, Sungkyunkwan University School of Medicine, Seoul, Korea; ^6^ Samsung Genome Institute, Samsung Medical Center, Seoul, Korea; ^7^ Department of Surgery, Samsung Medical Center, Sungkyunkwan University School of Medicine, Seoul, Korea; ^8^ Department of Pathology, Sanggye Paik Hospital, Inje University College of Medicine, Seoul, Korea; ^9^ Current address: Department of Pathology, Kangnam Sacred Heart Hospital, Hallym University, Seoul, Korea

**Keywords:** high-throughput nucleotide sequencing, gastrointestinal stromal tumor, exome, transcriptome

## Abstract

Gastrointestinal stromal tumors (GISTs) are the most common mesenchymal tumors of the gastrointestinal tract. We sequenced nine exomes and transcriptomes, and two genomes of GISTs for integrated analyses. We detected 306 somatic variants in nine GISTs and recurrent protein-altering mutations in 29 genes. Transcriptome sequencing revealed 328 gene fusions, and the most frequently involved fusion events were associated with *IGF2* fused to several partner genes including *CCND1*, *FUS*, and *LASP1*. We additionally identified three recurrent read-through fusion transcripts: *POLA2*-*CDC42EP2*, *C8orf42*-*FBXO25*, and *STX16*-*NPEPL1*. Notably, we found intragenic deletions in one of three exons of the *VHL* gene and increased mRNAs of *VEGF*, *PDGF-β*, and *IGF-1/2* in 56% of GISTs, suggesting a mechanistic link between *VHL* inactivation and overexpression of hypoxia-inducible factor target genes in the absence of hypoxia. We also identified copy number gain and increased mRNA expression of *AMACR*, *CRIM1*, *SKP2*, and *CACNA1E*. Mapping of copy number and gene expression results to the KEGG pathways revealed activation of the JAK-STAT pathway in small intestinal GISTs and the MAPK pathway in wild-type GISTs. These observations will allow us to determine the genetic basis of GISTs and will facilitate further investigation to develop new therapeutic options.

## INTRODUCTION

Gastrointestinal stromal tumors (GISTs) arise from the mesenchymal tissue of the gastrointestinal tract or, rarely, from intra-abdominal soft tissue [1]. Most GISTs harbor gain-of-function mutations in *KIT* (75-80%) or *PDGFRA* (∼10%) [2, 3]. The discovery of these activating mutations has led to the clinical use of the tyrosine kinase inhibitor imatinib mesylate in patients with advanced or metastatic GISTs [4, 5].

Tumor response to imatinib varies by primary genotype. For example, *KIT* exon 9 mutant or wild-type GISTs show a diminished response to imatinib compared with *KIT* exon 11 mutants, and GISTs with the *PDGFRA* D842V mutation show no response [6-8]. Moreover, secondary mutations in patients with long-term imatinib treatment are associated with tumor resistance [9]. Several clinical trials using novel agents that either target the *KIT* receptor directly through a different mechanism or target an alternative pathway are currently underway [10]. The identification of additional genetic events in GISTs will facilitate the development of effective targeted therapies for patients showing limited response to imatinib therapy.

GISTs have distinct gene expression profiles and clinical behavior depending on their genotype and location [11]. Specific chromosomal aberrations also correlate well with anatomic site and biologic behavior [12, 13]. Mutations in *BRAF*, *RAS*, or in the genes of the succinate dehydrogenase (SDH) complex, as well as overexpression of IGF1R, have been described as possible initial molecular events in a subset of wild-type GISTs [14-17].

In the last several years, next-generation sequencing studies have characterized the molecular landscape of diverse cancer types and have led to dramatic advances in cancer genomics [18]. In this study, the whole-genome, exome, and transcriptome of nine GISTs were sequenced and integrated with clinicopathologic features.

## RESULTS AND DISCUSSION

### Somatic mutations

**Figure 1:**
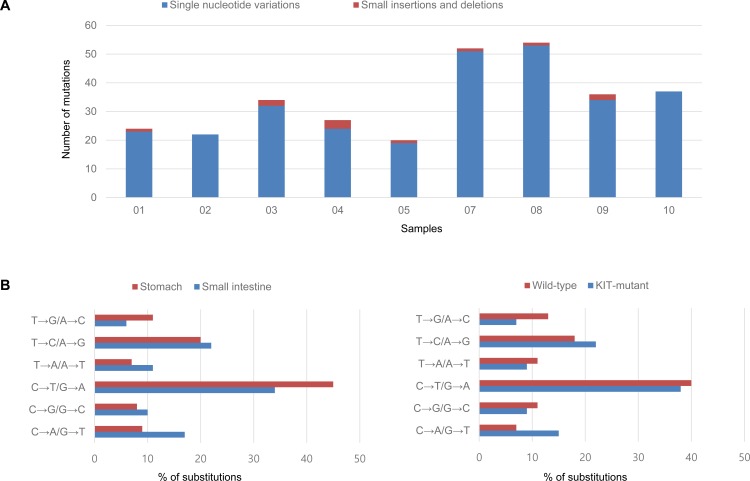
Mutation frequencies in each GIST sample (**A**) and spectra according to tumor location and KIT mutation status (**B**)

The clinicopathologic features of the analyzed GISTs are summarized in Table 1. Exome sequencing revealed 306 somatic variants (mean, 34; range, 20-54) including 172 missense, 13 nonsense, 3 stop codon loss, 16 splice-site, 11 small insertion and deletions (indels), and 90 synonymous mutations (Figure 1). Wild-type GISTs showed relatively lower mutation frequencies compared to *KIT* mutants (mean, 23 vs. 37; range, 22-24 vs. 20-54). However, their mutational signatures were similar, with the most frequent types being C>T/G>A transitions.

**Table 1:** Clinicopathologic data of nine gastrointestinal stromal tumors

Patient	Sample	Tumor location	Size (cm)	Mitotic rate (per 5 mm^2^)	Risk of progression	*KIT* & *PDGFRA* mutation	Recurrence or metastasis	Clinical reponse to imatinib
01	01	stomach	7	2	low	wild-type	no	no treatment
02	02	stomach	6	1	Low	wild-type	no	no treatment
03	03	stomach	6	6	High	*KIT* exon 11 deletion	yes	Y
04	04	stomach	12	9	High	*KIT* exon 13 missense (K642E)	yes	Y
05	05	stomach	6	3	low	*KIT* exon 11 missense (V559A)	no	no treatment
07	07	small intestine (primary)	12	10	high	*KIT* exon 17 missense (N822Y)	yes	N
	08	small intestine (recurrent)	9	7				
08	09	small intestine	9	18	high	*KIT* exon 11 deletion	yes	N
09	10	small intestine	11	8	high	*KIT* exon 9 insertion	yes	Y

Y, stable disease or partial reponse; N, progressive disease

**Figure 2:**
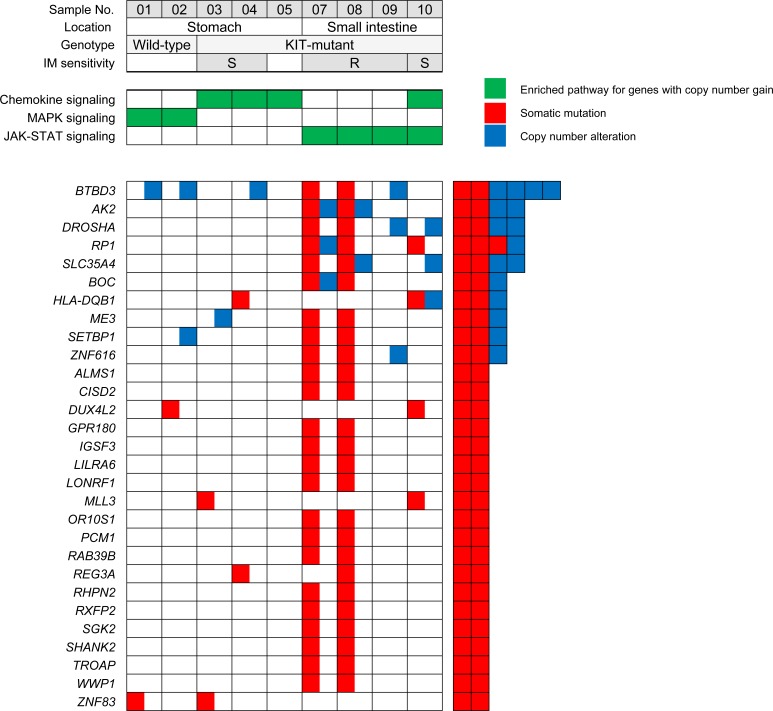
Recurrently mutated genes identified by whole-exome sequencing and the enriched KEGG pathways for genes altered by copy number gain in GISTs (IM, imatinib mesylate; S, sensitive; R, resistant)

There were 199 protein-altering somatic mutations in 160 genes. Among them, 29 genes were mutated in two or more samples (Figure 2 and Supplemental Table 1). All mutations except one were covered by sequence reads in the transcriptome analysis, and 141 (71%) demonstrated evidence of expression as defined by reads per kilobase per million (RPKM) ≥1. These also included two recurrently mutated genes (*HNRNPCL1* and *USP8*) identified in a prior study by targeted exome sequencing of 13 GISTs [19]. Cell adhesion was the most significantly enriched biological process among the mutated genes (DAVID Bioinformatics Resource 6.7). Novel mutations identified in this study include the histone methyltransferase gene *MLL3* (sample No. 3 and 10) and *EZH2*, a chromatin regulator gene found in an imatinib-resistant recurrent tumor (sample No. 8). *EZH2* mutations have been previously reported as a recurrent abnormality in atypical chronic myeloid leukemia [20, 21]. Several genes found mutated in wild-type GISTs include *ALPK2*, *DHDH*, *HERC2*, *PYGM*, *TRPC5*, *USP8*, and *ZNF83*, and they showed a score ≥1 by the oncogenic gene ranker (http://cbio.mskcc.org/tcga-generanker/).

In direct comparison of a primary and recurrent pair from the same patient (sample No. 7 and 8), many of the original mutations in the primary tumor were propagated in the recurrent tumor akin to the results of previous studies of melanoma and breast cancer [22, 23]. In this patient, a *WWP1* mutation was identified. This protein is known to affect the protein stability of various oncogenes (such as *ERBB4*) [24] and has been shown to be recurrently mutated in liver cancers [25]. A mutation in *SETBP1*, a newly discovered oncogene present in atypical chronic myeloid leukemias and related diseases [21, 26], was also observed. Although some mutations (*FAM153A*, *FOXP1*, *GGT1*, and *IRAK1*) were found only in the primary GIST, mutations in *EZH2*, *GBP7*, *HNRNPCL1*, *MAX*, *PLIN4*, *PLXNA2*, *PLXNB3*, *PSPH*, *REG3A*, *TXLNG*, and *XIRP2* were present only in the recurrence.

The *CASP7*, *EZH2*, and *ZNF430* mutations were confirmed by Sanger sequencing, and 50 additional malignant GISTs were tested for these mutations. However, no further mutations were found, indicating these are likely passenger or rare events in GISTs. Furthermore, a thorough search was performed for possible germline mutations in *KIT*, *PDGFRA*, *NF1*, and *SDH* subunits. One patient (sample No. 2) had a germline *SDHB* mutation (p.S163F) detected by exome sequencing. The patient had a wild-type GIST at the age of 35 with no prior family history of paraganglioma. This patient′s GIST showed SDHB protein expression loss by immunohistochemistry (Sigma-Aldrich, St ouis, MO, USA; 1:800). Previous gene expression analyses also showed a lower mRNA level of *SDHB* in this case compared to *KIT*-mutant GISTs [27].

### Gene fusions

**Figure 3:**
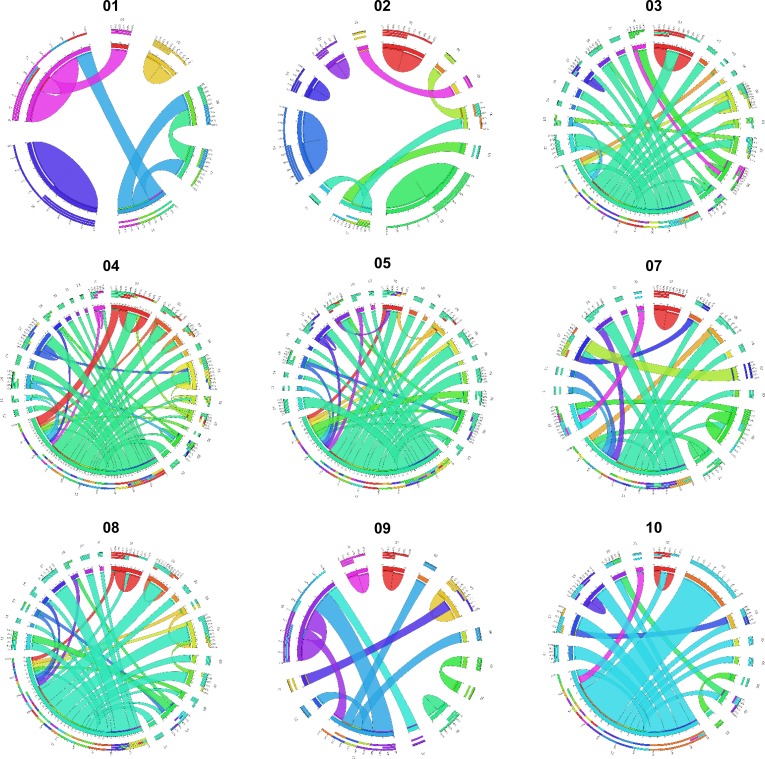
Gene fusions detected in nine GISTs displayed as Circos plots The width of the bands is proportion to the number of fusion events between two chromosomes (Read-through transcripts are not shown).

In this study, 328 gene fusion events were identified in nine GISTs by transcriptome analyses selected by the criteria described in the method section. The plots generated using the Circos Table Viewer for each case are provided in Figure 3. Gastric *KIT*-mutant GISTs (mean, 82.0; range, 68-109) expressed more frequent fusion transcripts than small intestinal (mean, 49.5; range, 40-67) or wild-type GISTs (mean, 33.0; range, 25-41) (Table 2). Of the fusion transcripts, 228 were ‘private′ (i.e., identified in only one tumor sample) at a range of 10 to 63 per tumor, and 100 events were detected in 2 or more tumors. Chromosome aberrations, in particular translocations and their corresponding gene fusions, have an important role in the initial steps of tumorigenesis. However, the biological and clinical impact of gene fusions in the more common solid tumor types has been less appreciated and most fusion genes were found from hematological cancers, sarcomas, and prostate cancer [28]. In this study, we first identified that gene fusion events are frequent in GISTs.

**Table 2:** Distribution of putative fusion transcripts in nine GIST samples

Sample	Private fusions			Recurrent fusions		
	Fusions per tumor	Number of genes	Read-through transcripts	Fusions per tumor	Number of genes	Read-through transcripts
01	10	18	3	15	29	11
02	22	41	11	19	37	14
03	30	41	7	38	64	19
04	63	88	17	46	77	23
05	30	39	7	39	57	15
07	22	37	9	29	48	15
08	24	32	4	43	63	14
09	14	27	5	26	49	20
10	13	20	3	27	43	11

Among the recurrent fusion transcripts, 13 and 11 were unique to gastric *KIT*-mutant and small intestinal tumors, respectively, but none of them were exclusively found in wild-type GISTs. A large number of fusion partner genes were located on chromosomes 1, 5, 9, 11, and 19. Although it is difficult to make any rigorous conclusions with respect to tumor specific distribution of fusion transcripts, chromosomes 4, 15, and 16 seem to be hot spots for gastric GISTs (Supplemental Table 2). These regions are well known to undergo copy number alterations (CNAs) in GISTs [2, 27]. Our findings support that fusion transcripts are functionally related to the genotype or location of GISTs [29]. Thus, establishing the role of these participating genes will provide important insights into the biology of GIST tumorigenesis.

In this study, the focus was narrowed to the fusion genes that have been reported to take part in translocations based on the Mitelman Database of Chromosome Aberrations (Table 3) [30]. One of the most frequently involved genes identified was *IGF2*, which fused to a number of different partners. Some of the partner genes (such as *EPS15*, *CCND1*, *LASP1*, *FUS*, and *HNRNPA2B1*) have been previously found to be involved in oncogenic fusions in other cancers including leukemias and lymphomas (COSMIC; http://cancer.sanger.ac.uk/cancergenome/projects/census). Additionally, three highly recurring gene fusions were also identified, including *POLA2*-*CDC42EP2* (n=7, 78% of cases), *C8orf42*-*FBXO25* (n=4), and *STX16*-*NPEPL1* (n=3). All three were read-through transcripts, which can be another mechanism of tumorigenesis [31] and may play a functional role in GISTs by potentially resulting in additional protein variation and changes in gene regulation. The *POLA2-CDC42EP2* fusion has been reported in bladder cancer cell line [32], and the fusion transcript involving *STX16* was previously reported in acute myeloid leukemia [33]. Using the tissue expression information from the ConjoinG database [34], *STX16-NPEPL1* was previously confirmed and found to be expressed in cancer tissues. This gene fusion was subsequently validated by reverse transcription polymerase chain reaction (RT-PCR) and Sanger sequencing (Supplemental Figure 1). It is also expected that highly expressed fusion genes are more important than those with low expression levels [35]. Eleven candidate fusions, including *STX16-NPEPL1*, had a higher expression level (>2-fold change compared to mean RPKM value) of one or both constituent partner genes (shown in Table 3), suggesting that the fusion events are linked to overexpression of the genes [30].

**Table 3:** Candidate fusion transcripts in gastrointestinal stromal tumors

Sample	Tool	5′			3′			Read-through
		Gene	Chromosome	FC* ≥ 2	Gene	Chromosome	FC* ≥ 2	
01	DF / CS	***POLA2***	11		*CDC42EP2*	11		Yes
01	DF	*IGF2*	11		***ACTB***	7		
01	CS	***STX16***	20		*NPEPL1*	20	2.10	Yes
02	DF / CS	***POLA2***	11		*CDC42EP2*	11		Yes
02	DF	***FBXO25***	8		***BET1L***	11		
03	DF	***EPS15***	1		*OSBPL9*	1		
03	DF / CS	***POLA2***	11		*CDC42EP2*	11		Yes
03	DF	*IGF2*	11		***SEPT9***	17		
03	DF	***GAPDH***	12		*IGF2*	11		
03	DF	*C8orf42*	8		***FBXO25***	8		Yes
03	DF	*IGF2*	11		***CCND1***	11	2.77	
03	CS	*LYPLA1*	8		***TCEA1***	8		
03	CS	*FXYD6-FXYD2*	11		***DSCAML1***	11	2.00	Yes
04	DF	*IGF2*	11	2.18	***EPC1***	10		
04	DF	*IGF2*	11	2.18	***SQSTM1***	5		
04	DF	*IGF2*	11	2.18	***TOP2B***	3		
04	DF	*IGF2*	11	2.18	***KIT***	4	2.59	
04	DF	***HNRNPA2B1***	7		*PCBD2*	5		
04	DF	***UBR4***	1	3.36	*IGF2*	11	2.18	
04	DF	*C8orf42*	8		***FBXO25***	8		Yes
04	DF / CS	***POLA2***	11		*CDC42EP2*	11		Yes
04	CS	*FXYD6-FXYD2*	11		***DSCAML1***	11	3.14	Yes
05	DF	***POLA2***	11		*CDC42EP2*	11		Yes
05	DF	*IGF2*	11		***LASP1***	17		
05	DF	*IGF2*	11		***FUS***	16		
05	CS	***STX16***	20		*NPEPL1*	20		Yes
07	DF / CS	*P2RY6*	11	5.27	***ARHGEF17***	11		Yes
07	CS	*CTSL3*	9	4.48	***GABBR2***	9		
08	DF	*IGF2*	11		***STAT5B***	17		
08	DF	***HNRNPA2B1***	7		*PCBD2*	5		
08	DF	*C8orf42*	8		***FBXO25***	8		Yes
08	DF	*CTSL1*	9		***GAPDH***	12		
08	DF	*IGF2*	11		***TNPO1***	5		
08	DF	***KIT***	4		*IGF2*	11		
08	DF	***KIT***	4		*PCBD2*	5		
08	CS	***POLA2***	11		*CDC42EP2*	11		Yes
09	DF	*C8orf42*	8		***FBXO25***	8		Yes
09	CS	***POLA2***	11		*CDC42EP2*	11		Yes
09	CS	***STX16***	20		*NPEPL1*	20		Yes
09	DF / CS	***RALGPS1***	9		*LRSAM1*	9		
10	DF	*IGF2*	11		***RBMS1***	2		

*Fold change (FC) represents the difference in RPKM values between a sample and the mean. Bold font indicates the genes listed in the Mitelman database (DF, defuse; CS, ChimeraScan).

### Copy number alterations and gene expression

Profiling of CNAs using exome sequencing identified recurrent gains of 5p (n=5), 5q (n=4), and 17q (n=2) and losses of 14q (n=7), 22q (n=6), 1p (n=4), 15q (n=4), and 18q (n=3) (Supplemental Figure 2). CNAs showed a site-dependent pattern, including higher frequency of losses at 1p and 15q (100% vs. 0%) in small intestinal vs. gastric GISTs. Moreover, all five cases (sample No. 3, 4, 7, 9, and 10) with subsequent metastasis had losses of 22q, four of which harbored additional gains on 5p. The patterns of broad cytogenetic gain and loss were consistent with the results of previous studies [12, 13, 36], indicating that the tumors in this series have the cardinal chromosomal changes ascribed to GISTs′ development and progression. According to the threshold defined in the method section, there were a total of 4484 and 9924 regions of gain and loss, respectively, at the gene level across the nine samples. By comparisons of CNAs at each gene locus identified in two wild-type GISTs, the concordance rate between whole-genome and exome sequencing was 96.7%. The mean number of CNAs in wild-type GISTs was 1111.5, compared to 1740.7 (range, 1187-2447) in *KIT*-mutant tumors. The number of CNAs in a sample approximately correlated with the number of protein-altering somatic mutations, reflecting their accumulated genetic alterations (Supplemental Figure 2). Ten genes (*AK2*, *BOC*, *BTBD3*, *DROSHA*, *HLA-DQB1*, *ME3*, *RP1*, *SETBP1*, *SLC35A4*, and *ZNF616*) harboring recurrent mutations were also located in regions of CNAs (Figure 2), suggesting that they might be implicated in the development or progression of GISTs.

**Figure 4:**
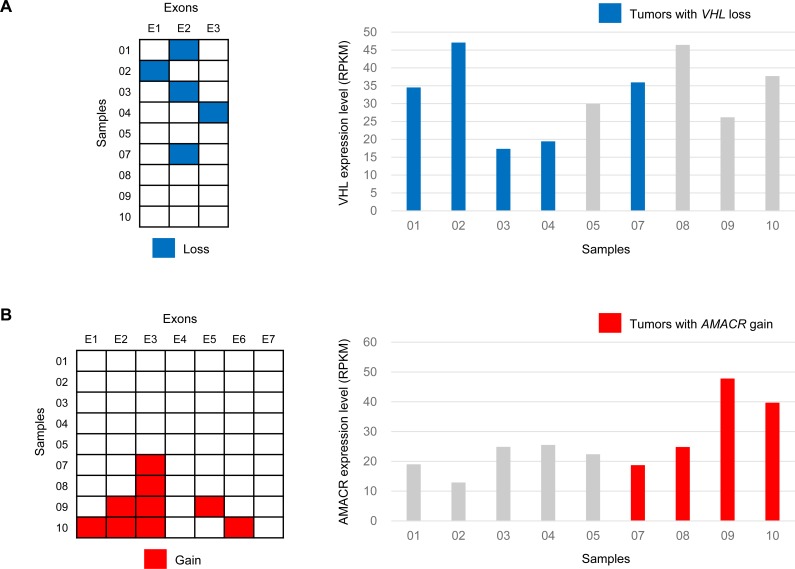
Exon-level copy number changes and overall gene expression in tumor samples with VHL loss (**A**) and AMACR gain (**B**)

For the analysis of CNAs that overlap with known cancer-related genes, the Cancer Census genes were classified into two categories (dominant and recessive) according to their annotations in the database. Of the dominant genes, frequent copy number gains were found in *IL7R* and *PDGFRB* (n=4, 44% of cases), followed by *EBF1*, *LIFR* (n=3), *BCL6*, and *HOXC13* (n=2). Among the recessive group genes, the most recurrent losses were seen in *CHEK2* (n=6, 67% of cases), followed by *EP300*, *SMARCB1*, *VHL* (n=5), *BUB1B*, *PMS2*, *SBDS* (n=4), *BLM*, *SDHB* (n=3), *BRCA1*, *FANCA*, and *FANCD2* (n=2). High-level losses of *CHEK2*, encoding a cell cycle regulator and putative tumor suppressor protein, were found in high-risk but not in low-risk GISTs. Interestingly, deletions at the *VHL* locus were found resulting in loss of one of three exons in 56% of the cases (Figure 4). Further analysis of copy number and exon-specific RNA sequencing revealed slightly lower *VHL* mRNA expression in *VHL*-deleted cases compared to non-deleted GISTs (mean RPKM, 30.9 vs. 35.1), however, there was no statistical significance. It is presumed that heterozygous loss of one exon has little effect on overall gene expression. However, in *VHL*-deleted GISTs, although hypoxia-inducible factor-1alpha (HIF-1α) was not always increased, mRNA levels of *VEGF* (4.2-fold change), *PDGF-β* (3.9-fold change), and *IGF-1/2* (2.2 and 1.7-fold change, respectively) were increased, suggesting a possible mechanistic link between *VHL* inactivation and overexpression of HIF-1α target genes in the absence of hypoxia. A number of agents that target these growth factors or their receptors are currently undergoing clinical trials, and the *VHL* status might be potentially important in predicting therapeutic response. In addition, it will be important to identify alternative *VHL* function independent of *HIF-1α* regulation [37].

The outlier genes, characterized by general low expression with marked overexpression in a fraction of the samples, are of great interest because the aberrant expression may arise as a result of their involvement in underlying recurrent genetic changes [38, 39]. In this study, 400 outlier genes were identified with copy number gain in nine GISTs. Among them, 13 (*AMACR*, *ANXA6*, *BTNL9*, *C1QTNF3-AMACR*, *DIAPH1*, *FAM153A*, *GRIA1*, *IL9*, *NMUR2*, *NXPH4*, *RAB6B*, *TPPP*, and *UGT3A2*) were recurrent in more than one GIST. In addition, *CRIM1* copy number gain with mRNA overexpression was identified in a wild-type GIST (sample No. 1). Its overexpression has been reported in an imatinib-resistant GIST cell line and in multidrug-resistant myeloid leukemia cells [40, 41]. *AMACR* has shown potential as a diagnostic marker and therapeutic target for prostate cancer. *AMACR* copy number gain (amplification) was observed in four GISTs, and it also showed significantly increased mRNA expression in two high-risk GISTs (Figure 4). By reanalysis of prior array comparative genomic hybridization data [27], a significant gain (log_2_-ratio > 0.3) at the *AMACR* locus was identified in six of 32 samples (19%). In all amplified cases, AMACR protein overexpression was confirmed by immunohistochemistry (clone 13H4, Dako) with 100% correlation (Supplemental Figure 3). It was found that AMACR overexpression is caused by DNA copy number gain in a subset of GISTs, and it is noteworthy that increased mRNA expression directly translates into protein overexpression. AMACR immunohistochemistry was then performed in additional 60 low-risk and 32 high-risk GISTs, as well as in other neoplasms in the differential diagnosis of GIST including 22 fibromatoses, 10 melanomas, and 10 malignant peripheral nerve sheath tumors. AMACR was positive in five (8%) low-risk GISTs, three (9%) high-risk GISTs, 0 fibromatosis, one (10%) melanoma, and two (20%) malignant peripheral nerve sheath tumors. One relevant dataset from the NCBI GEO database (Profile ID: GDS1209) compared AMACR expression in two GISTs and normal tissue samples from 15 different sites, and we could find any significant differences. However, *AMACR* amplification and overexpression in primary GISTs driving cell proliferation has been recently reported during preparation of this manuscript [42].

Among the 77 genes with previous evidence for amplification and consequent overexpression [43], recurrent gains of *SKP2* (sample No. 7, 9, and 10) and *CACNA1E* (sample No. 7 and 9) were found. The mean RPKM values of the *SKP2* and *CACNA1E* genes were higher in cases with copy number gain compared to others (49.9 vs. 26.8 and 8.6 vs. 1.2, respectively). SKP2 overexpression is associated with a poor prognosis in various cancers, including soft tissue sarcoma and GISTs [44-46]. It has been also reported that imatinib induces GIST cell quiescence through the APC/CDH1–SKP2–p27 (Kip1) signaling axis [46].

### Integrative pathway analysis

First, a pathway enrichment analysis of the genes with copy number gains from the exome sequencing data was performed, and several overrepresented KEGG pathways were identified. The chemokine signaling pathway altered in both gastric and small intestinal GISTs is capable of activating diverse downstream signaling pathways (including the MAPK, PI3K-Akt, and JAK-STAT pathways). The MAPK signaling pathway was enriched only in gastric wild-type GISTs, while the JAK-STAT pathway seemed to be more associated with small intestinal GISTs irrespective of response to imatinib (Figure 2). The genes mapped to the corresponding pathways are depicted in Supplemental Figure 4.

**Figure 5:**
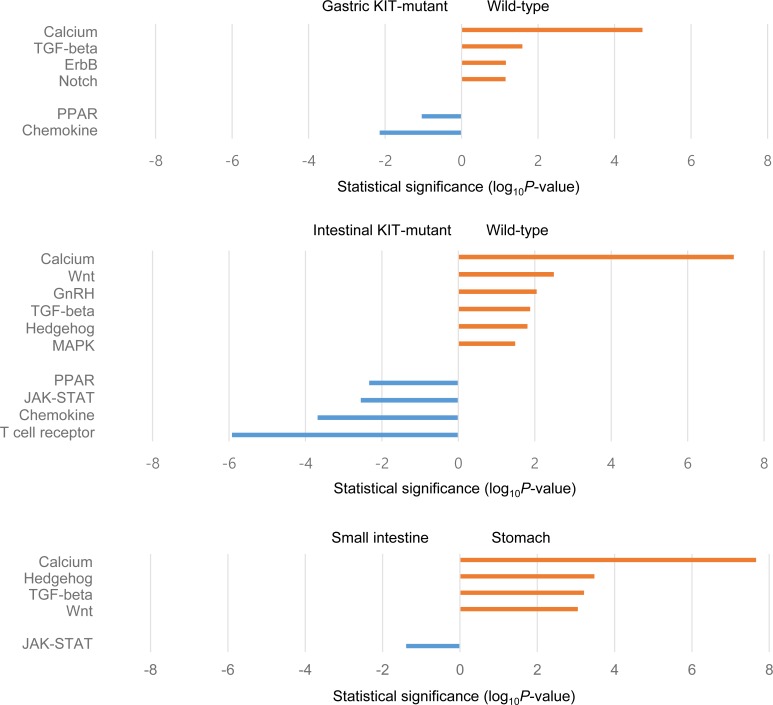
Selected KEGG pathways enriched by differentially expressed genes between groups of samples

To investigate gene expression differences in nine GISTs, the transcriptome sequencing data were subjected to multidimensional scaling (MDS) analysis. There was a clear separation according to tumor location (gastric vs. small intestinal) and genotype (wild-type vs. *KIT*-mutant). It was found that imatinib-sensitive GISTs were loosely clustered and not distinct from imatinib-resistant tumors (supplemental Figure 5). Comparisons of gene expression between wild-type and *KIT*-mutant GISTs, as well as between gastric and intestinal tumors, were then performed. In line with the copy number results, KEGG analysis revealed significant differences in the level of pathway activation between tumor subtypes (Figure 5). Specifically, tumors in the small intestine showed preferential activation of the JAK-STAT pathway, and wild-type GISTs appeared to also use the MAPK signaling pathway. The two pathway diagrams are provided in Supplemental Figure 4 with overexpressed genes indicated. When the RPKM values were compared between tumor subtypes for the pathway component genes previously defined by Lui et al. [47], 10 (71%) and 9 (60%) genes belonging to the MAPK and the JAK-STAT pathway were overexpressed in gastric wild-type and small intestinal *KIT*-mutant GISTs, respectively (Supplemental Figure 6). Thus, these results suggest that activation of different signaling pathways may correlate to tumor development, progression, and response to treatment.

In conclusion, the present study provides the most comprehensive catalog of genomic alteration in GISTs to date, leading to the discovery of multiple previously unreported mutations and candidate gene fusions that can be prioritized for further investigation. The genetic properties of GISTs are heterogeneous, and therefore present a challenge in the era of targeted therapy. Although functional mechanisms are not provided in this study, frequent fusion events and genetic alterations of *VHL* and *AMACR* may contribute to GIST pathogenesis. The molecular and pathway signatures identified here will facilitate the development of tumor-specific targeted therapies for GIST patients.

## MATERIALS AND METHODS

### Patient and sample characteristics

The study consisted of nine primary or metastatic GISTs from eight patients (Table 1). Two were wild-type for *KIT* (exons 9, 11, 13, and 17) and *PDGFRA* (exons 12, 14, and 18). All patients underwent complete surgical resection of the primary tumor without prior imatinib therapy. Among them, five patients (No. 3, 4, 7, 8, and 9) showed recurrence or metastasis of disease and subsequently received imatinib therapy. Three had a partial response and one patient (No. 7) showed disease progression requiring an additional resection. All patients were alive with a median follow-up of 90 months. Written informed consent was obtained before sample collection. The institutional ethics review board of Samsung Medical Center approved this study.

### Whole-genome/exome sequencing (WGS/WES) and data analysis

Genomic DNA was extracted from snap-frozen tumors with matching peripheral blood samples using QIAamp DNA Mini kits (Qiagen), and was subjected to exome capture with SureSelect Human All Exon 50Mb kit (Agilent Technologies) per the manufacturers′ instructions. Both WGS and WES were carried out on the Illumina HiSeq 2000 platform in 100-bp paired-end reads. Sequencing statistics are provided in Supplemental Table 3. Obtained FASTQ files were aligned to the human reference genome (GRCh37/hg19) using Burrows-Wheeler Aligner [48].

The GATK UnifiedGenotyper [49], as well as an in-house pipeline, were used to detect single-nucleotide variants and small indels from the WES data. The observed read counts were modeled with beta distributions to calculate the probability of difference in variant allele fraction between matched tumor and normal samples. The SIFT algorithm was used to predict the putative effect of each nonsynonymous mutation on protein function [50]. Control-FREEC [51] and CONTRA [52] were used to infer copy number changes from WGS and WES data, respectively, and the following criteria were applied for gene-level copy number estimation: 1) segments with log_2_-ratio > 0.3 and < −0.3 were designated as regions of gain and loss, respectively; 2) at least 20% of the exons in a gene must have a significant CONTRA call. Structural variations of two wild-type tumors were also analyzed using SVDetect [53].

### Whole-transcriptome sequencing (RNA-seq) and data analysis

RNA was extracted using the RNeasy Mini kit (Qiagen), and mRNA libraries were prepared using the TruSeq RNA Sample Preparation kit according to the manufacturer′s protocol (Illumina). Paired-end 101-bp reads were generated using the Illumina HiScanSQ platform and aligned against the human genome (hg19) using TopHat [54]. Detailed RNA-seq metrics are presented in Supplemental Table 3.

Gene expression levels were measured in RPKM [55]. The outlier sum statistic was applied for analysis of outlier gene expression profiles [56], and differentially expressed genes between the groups were identified by fold-change filtering and using edgeR [57]. Gene fusions were predicted by combining the results of deFuse [58] and ChimeraScan [59] algorithms. It was required that each candidate fusion transcript has at least two distinct read pairs and junction-spanning reads. To validate fusion candidates on the DNA level, PCR primers were designed to flank the predicted breakpoints by Primer5.0. PCR products were gel excised and then sequenced on a 3130xl DNA analyzer (Applied Biosystems).

### Data access

The sequencing data have been submitted to the NCBI Sequence Read Archive (SRA; http://www.ncbi.nlm.nih.gov/sra/) under accession numbers SRP036055, SRP042250, and SRP041531.

## SUPPLEMENTARY MATERIAL FIGURES AND TABLES



## References

[1] Miettinen M, Lasota J (2011). Histopathology of gastrointestinal stromal tumor. J Surg Oncol.

[2] Corless CL, Barnett CM, Heinrich MC (2011). Gastrointestinal stromal tumours: origin and molecular oncology. Nature reviews Cancer.

[3] Joensuu H, Hohenberger P, Corless CL (2013). Gastrointestinal stromal tumour. Lancet.

[4] Demetri GD, von Mehren M, Blanke CD, Van den Abbeele AD, Eisenberg B, Roberts PJ, Heinrich MC, Tuveson DA, Singer S, Janicek M, Fletcher JA, Silverman SG, Silberman SL, Capdeville R, Kiese B, Peng B (2002). Efficacy and safety of imatinib mesylate in advanced gastrointestinal stromal tumors. N Engl J Med.

[5] Joensuu H, Roberts PJ, Sarlomo-Rikala M, Andersson LC, Tervahartiala P, Tuveson D, Silberman S, Capdeville R, Dimitrijevic S, Druker B, Demetri GD (2001). Effect of the tyrosine kinase inhibitor STI571 in a patient with a metastatic gastrointestinal stromal tumor. N Engl J Med.

[6] Corless CL, Schroeder A, Griffith D, Town A, McGreevey L, Harrell P, Shiraga S, Bainbridge T, Morich J, Heinrich MC (2005). PDGFRA mutations in gastrointestinal stromal tumors: frequency, spectrum and *in vitro* sensitivity to imatinib. Journal of clinical oncology.

[7] Heinrich MC, Corless CL, Demetri GD, Blanke CD, von Mehren M, Joensuu H, McGreevey LS, Chen CJ, Van den Abbeele AD, Druker BJ, Kiese B, Eisenberg B, Roberts PJ, Singer S, Fletcher CD, Silberman S (2003). Kinase mutations and imatinib response in patients with metastatic gastrointestinal stromal tumor. Journal of clinical oncology.

[8] Debiec-Rychter M, Dumez H, Judson I, Wasag B, Verweij J, Brown M, Dimitrijevic S, Sciot R, Stul M, Vranck H, Scurr M, Hagemeijer A, van Glabbeke M, van Oosterom AT (2004). Use of c-KIT/PDGFRA mutational analysis to predict the clinical response to imatinib in patients with advanced gastrointestinal stromal tumours entered on phase I and II studies of the EORTC Soft Tissue and Bone Sarcoma Group. Eur J Cancer.

[9] Heinrich MC, Corless CL, Blanke CD, Demetri GD, Joensuu H, Roberts PJ, Eisenberg BL, von Mehren M, Fletcher CD, Sandau K, McDougall K, Ou WB, Chen CJ, Fletcher JA (2006). Molecular correlates of imatinib resistance in gastrointestinal stromal tumors. J Clin Oncol.

[10] Lamba G, Ambrale S, Lee B, Gupta R, Rafiyath SM, Liu D (2012). Recent advances and novel agents for gastrointestinal stromal tumor (GIST). J Hematol Oncol.

[11] Antonescu CR, Viale A, Sarran L, Tschernyavsky SJ, Gonen M, Segal NH, Maki RG, Socci ND, DeMatteo RP, Besmer P (2004). Gene expression in gastrointestinal stromal tumors is distinguished by KIT genotype and anatomic site. Clin Cancer Res.

[12] Wozniak A, Sciot R, Guillou L, Pauwels P, Wasag B, Stul M, Vermeesch JR, Vandenberghe P, Limon J, Debiec-Rychter M (2007). Array CGH analysis in primary gastrointestinal stromal tumors: cytogenetic profile correlates with anatomic site and tumor aggressiveness, irrespective of mutational status. Genes Chromosomes Cancer.

[13] Gunawan B, von Heydebreck A, Sander B, Schulten HJ, Haller F, Langer C, Armbrust T, Bollmann M, Gasparov S, Kovac D, Fuzesi L (2007). An oncogenetic tree model in gastrointestinal stromal tumours (GISTs) identifies different pathways of cytogenetic evolution with prognostic implications. J Pathol.

[14] Hostein I, Faur N, Primois C, Boury F, Denard J, Emile JF, Bringuier PP, Scoazec JY, Coindre JM (2010). BRAF mutation status in gastrointestinal stromal tumors. Am J Clin Pathol.

[15] Janeway KA, Kim SY, Lodish M, Nose V, Rustin P, Gaal J, Dahia PL, Liegl B, Ball ER, Raygada M, Lai AH, Kelly L, Hornick JL, O′Sullivan M, de Krijger RR, Dinjens WN (2011). Defects in succinate dehydrogenase in gastrointestinal stromal tumors lacking KIT and PDGFRA mutations. Proc Natl Acad Sci U S A.

[16] Miranda C, Nucifora M, Molinari F, Conca E, Anania MC, Bordoni A, Saletti P, Mazzucchelli L, Pilotti S, Pierotti MA, Tamborini E, Greco A, Frattini M (2012). KRAS and BRAF mutations predict primary resistance to imatinib in gastrointestinal stromal tumors. Clin Cancer Res.

[17] Tarn C, Rink L, Merkel E, Flieder D, Pathak H, Koumbi D, Testa JR, Eisenberg B, von Mehren M, Godwin AK (2008). Insulin-like growth factor 1 receptor is a potential therapeutic target for gastrointestinal stromal tumors. Proc Natl Acad Sci U S A.

[18] Shyr D, Liu Q (2013). Next generation sequencing in cancer research and clinical application. Biol Proced Online.

[19] Schoppmann SF, Vinatzer U, Popitsch N, Mittlbock M, Liebmann-Reindl S, Jomrich G, Streubel B, Birner P (2013). Novel Clinically Relevant Genes in Gastrointestinal Stromal Tumors Identified by Exome Sequencing. Clin Cancer Res.

[20] Ernst T, Chase AJ, Score J, Hidalgo-Curtis CE, Bryant C, Jones AV, Waghorn K, Zoi K, Ross FM, Reiter A, Hochhaus A, Drexler HG, Duncombe A, Cervantes F, Oscier D, Boultwood J (2010). Inactivating mutations of the histone methyltransferase gene EZH2 in myeloid disorders. Nat Genet.

[21] Piazza R, Valletta S, Winkelmann N, Redaelli S, Spinelli R, Pirola A, Antolini L, Mologni L, Donadoni C, Papaemmanuil E, Schnittger S, Kim DW, Boultwood J, Rossi F, Gaipa G, De Martini GP (2013). Recurrent SETBP1 mutations in atypical chronic myeloid leukemia. Nat Genet.

[22] Turajlic S, Furney SJ, Lambros MB, Mitsopoulos C, Kozarewa I, Geyer FC, Mackay A, Hakas J, Zvelebil M, Lord CJ, Ashworth A, Thomas M, Stamp G, Larkin J, Reis-Filho JS, Marais R (2012). Whole genome sequencing of matched primary and metastatic acral melanomas. Genome Res.

[23] Ding L, Ellis MJ, Li S, Larson DE, Chen K, Wallis JW, Harris CC, McLellan MD, Fulton RS, Fulton LL, Abbott RM, Hoog J, Dooling DJ, Koboldt DC, Schmidt H, Kalicki J (2010). Genome remodelling in a basal-like breast cancer metastasis and xenograft. Nature.

[24] Li Y, Zhou Z, Alimandi M, Chen C (2009). WW domain containing E3 ubiquitin protein ligase 1 targets the full-length ErbB4 for ubiquitin-mediated degradation in breast cancer. Oncogene.

[25] Fujimoto A, Totoki Y, Abe T, Boroevich KA, Hosoda F, Nguyen HH, Aoki M, Hosono N, Kubo M, Miya F, Arai Y, Takahashi H, Shirakihara T, Nagasaki M, Shibuya T, Nakano K (2012). Whole-genome sequencing of liver cancers identifies etiological influences on mutation patterns and recurrent mutations in chromatin regulators. Nat Genet.

[26] Fernandez-Mercado M, Pellagatti A, Di Genua C, Larrayoz MJ, Winkelmann N, Aranaz P, Burns A, Schuh A, Calasanz MJ, Cross NC, Boultwood J (2013). Mutations in SETBP1 are recurrent in myelodysplastic syndromes and often coexist with cytogenetic markers associated with disease progression. Br J Haematol.

[27] Lee EJ, Kang G, Kang SW, Jang KT, Lee J, Park JO, Park CK, Sohn TS, Kim S, Kim KM (2013). GSTT1 copy number gain and ZNF overexpression are predictors of poor response to imatinib in gastrointestinal stromal tumors. PLoS One.

[28] Mitelman F, Johansson B, Mertens F (2007). The impact of translocations and gene fusions on cancer causation. Nat Rev Cancer.

[29] Asmann YW, Necela BM, Kalari KR, Hossain A, Baker TR, Carr JM, Davis C, Getz JE, Hostetter G, Li X, McLaughlin SA, Radisky DC, Schroth GP, Cunliffe HE, Perez EA, Thompson EA (2012). Detection of redundant fusion transcripts as biomarkers or disease-specific therapeutic targets in breast cancer. Cancer Res.

[30] Edgren H, Murumagi A, Kangaspeska S, Nicorici D, Hongisto V, Kleivi K, Rye IH, Nyberg S, Wolf M, Borresen-Dale AL, Kallioniemi O (2011). Identification of fusion genes in breast cancer by paired-end RNA-sequencing. Genome Biol.

[31] Kim HP, Cho GA, Han SW, Shin JY, Jeong EG, Song SH, Lee WC, Lee KH, Bang D, Seo JS, Kim JI, Kim TY (2014). Novel fusion transcripts in human gastric cancer revealed by transcriptome analysis. Oncogene.

[32] Jia W, Qiu K, He M, Song P, Zhou Q, Zhou F, Yu Y, Zhu D, Nickerson ML, Wan S, Liao X, Zhu X, Peng S, Li Y, Wang J, Guo G (2013). SOAPfuse: an algorithm for identifying fusion transcripts from paired-end RNA-Seq data. Genome Biol.

[33] Wen H, Li Y, Malek SN, Kim YC, Xu J, Chen P, Xiao F, Huang X, Zhou X, Xuan Z, Mankala S, Hou G, Rowley JD, Zhang MQ, Wang SM (2012). New fusion transcripts identified in normal karyotype acute myeloid leukemia. PLoS One.

[34] Prakash T, Sharma VK, Adati N, Ozawa R, Kumar N, Nishida Y, Fujikake T, Takeda T, Taylor TD (2010). Expression of conjoined genes: another mechanism for gene regulation in eukaryotes. PLoS One.

[35] Liu C, Ma J, Chang CJ, Zhou X (2013). FusionQ: a novel approach for gene fusion detection and quantification from paired-end RNA-Seq. BMC Bioinformatics.

[36] El-Rifai W, Sarlomo-Rikala M, Andersson LC, Knuutila S, Miettinen M (2000). DNA sequence copy number changes in gastrointestinal stromal tumors: tumor progression and prognostic significance. Cancer Res.

[37] Kim WY, Kaelin WG (2004). Role of VHL gene mutation in human cancer. J Clin Oncol.

[38] Tomlins SA, Rhodes DR, Perner S, Dhanasekaran SM, Mehra R, Sun XW, Varambally S, Cao X, Tchinda J, Kuefer R, Lee C, Montie JE, Shah RB, Pienta KJ, Rubin MA, Chinnaiyan AM (2005). Recurrent fusion of TMPRSS2 and ETS transcription factor genes in prostate cancer. Science.

[39] Harvey RC, Mullighan CG, Wang X, Dobbin KK, Davidson GS, Bedrick EJ, Chen IM, Atlas SR, Kang H, Ar K, Wilson CS, Wharton W, Murphy M, Devidas M, Carroll AJ, Borowitz MJ (2010). Identification of novel cluster groups in pediatric high-risk B-precursor acute lymphoblastic leukemia with gene expression profiling: correlation with genome-wide DNA copy number alterations, clinical characteristics, and outcome. Blood.

[40] Mahadevan D, Cooke L, Riley C, Swart R, Simons B, Della Croce K, Wisner L, Iorio M, Shakalya K, Garewal H, Nagle R, Bearss D (2007). A novel tyrosine kinase switch is a mechanism of imatinib resistance in gastrointestinal stromal tumors. Oncogene.

[41] Prenkert M, Uggla B, Tidefelt U, Strid H (2010). CRIM1 is expressed at higher levels in drug-resistant than in drug-sensitive myeloid leukemia HL60 cells. Anticancer Res.

[42] Li CF, Chen LT, Lan J, Chou FF, Lin CY, Chen YY, Chen TJ, Li SH, Yu SC, Fang FM, Tai HC, Huang HY (2014). AMACR amplification and overexpression in primary imatinib-naive gastrointestinal stromal tumors: a driver of cell proliferation indicating adverse prognosis. Oncotarget.

[43] Santarius T, Shipley J, Brewer D, Stratton MR, Cooper CS (2010). A census of amplified and overexpressed human cancer genes. Nat Rev Cancer.

[44] Di Vizio D, Demichelis F, Simonetti S, Pettinato G, Terracciano L, Tornillo L, Freeman MR, Insabato L (2008). Skp2 expression is associated with high risk and elevated Ki67 expression in gastrointestinal stromal tumours. BMC Cancer.

[45] Oliveira AM, Okuno SH, Nascimento AG, Lloyd RV (2003). Skp2 protein expression in soft tissue sarcomas. J Clin Oncol.

[46] Liu Y, Perdreau SA, Chatterjee P, Wang L, Kuan SF, Duensing A (2008). Imatinib mesylate induces quiescence in gastrointestinal stromal tumor cells through the CDH1-SKP2-p27Kip1 signaling axis. Cancer Res.

[47] Lui VW, Hedberg ML, Li H, Vangara BS, Pendleton K, Zeng Y, Lu Y, Zhang Q, Du Y, Gilbert BR, Freilino M, Sauerwein S, Peyser ND, Xiao D, Diergaarde B, Wang L (2013). Frequent mutation of the PI3K pathway in head and neck cancer defines predictive biomarkers. Cancer Discov.

[48] Li H, Durbin R (2009). Fast and accurate short read alignment with Burrows-Wheeler transform. Bioinformatics.

[49] McKenna A, Hanna M, Banks E, Sivachenko A, Cibulskis K, Kernytsky A, Garimella K, Altshuler D, Gabriel S, Daly M, DePristo MA (2010). The Genome Analysis Toolkit: a MapReduce framework for analyzing next-generation DNA sequencing data. Genome Res.

[50] Kumar P, Henikoff S, Ng PC (2009). Predicting the effects of coding non-synonymous variants on protein function using the SIFT algorithm. Nat Protoc.

[51] Boeva V, Popova T, Bleakley K, Chiche P, Cappo J, Schleiermacher G, Janoueix-Lerosey I, Delattre O, Barillot E (2012). Control-FREEC: a tool for assessing copy number and allelic content using next-generation sequencing data. Bioinformatics.

[52] Li J, Lupat R, Amarasinghe KC, Thompson ER, Doyle MA, Ryland GL, Tothill RW, Halgamuge SK, Campbell IG, Gorringe KL (2012). CONTRA: copy number analysis for targeted resequencing. Bioinformatics.

[53] Zeitouni B, Boeva V, Janoueix-Lerosey I, Loeillet S, Legoix-ne P, Nicolas A, Delattre O, Barillot E (2010). SVDetect: a tool to identify genomic structural variations from paired-end and mate-pair sequencing data. Bioinformatics.

[54] Trapnell C, Pachter L, Salzberg SL (2009). TopHat: discovering splice junctions with RNA-Seq. Bioinformatics.

[55] Mortazavi A, Williams BA, McCue K, Schaeffer L, Wold B (2008). Mapping and quantifying mammalian transcriptomes by RNA-Seq. Nat Methods.

[56] Tibshirani R, Hastie T (2007). Outlier sums for differential gene expression analysis. Biostatistics.

[57] Robinson MD, McCarthy DJ, Smyth GK (2010). edgeR: a Bioconductor package for differential expression analysis of digital gene expression data. Bioinformatics.

[58] McPherson A, Hormozdiari F, Zayed A, Giuliany R, Ha G, Sun MG, Griffith M, Heravi Moussavi A, Senz J, Melnyk N, Pacheco M, Marra MA, Hirst M, Nielsen TO, Sahinalp SC, Huntsman D (2011). deFuse: an algorithm for gene fusion discovery in tumor RNA-Seq data. PLoS Comput Biol.

[59] Iyer MK, Chinnaiyan AM, Maher CA (2011). ChimeraScan: a tool for identifying chimeric transcription in sequencing data. Bioinformatics.

