# Supplementary material for: Integrated genomic analyses identify frequent gene fusion events and VHL inactivation in gastrointestinal stromal tumors
Source: Oncotarget. 2015 Mar 30;7(6):6538–51. doi: 10.18632/oncotarget.3731 (PMC4872731; doi:10.18632/oncotarget.3731)
Supplement: Supplementary file 1 [file oncotarget-07-6538-s001.pdf]

Integrated genomic analyses identify frequent gene fusion events and *VHL* inactivation in gastrointestinal stromal tumors

Supplementary Material

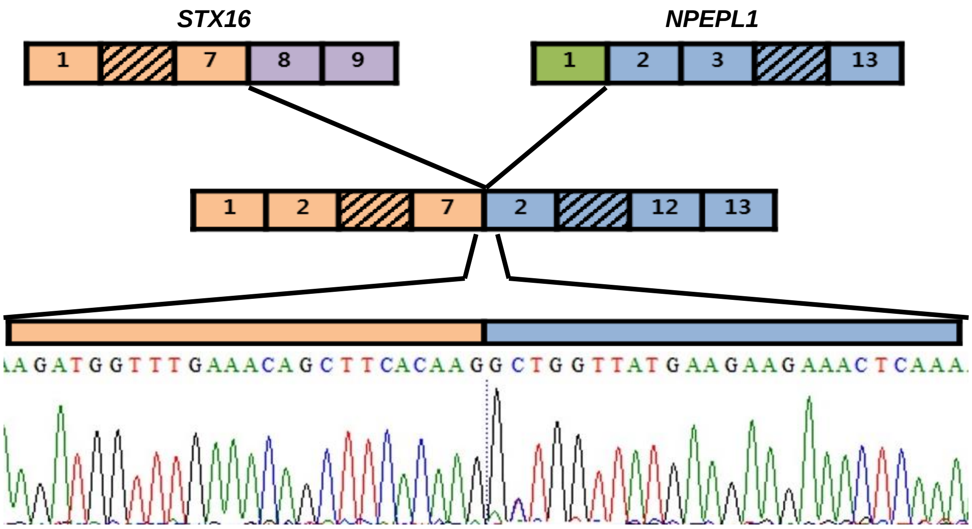

Figure S1: *STX16-NPEPL1* fusion identified by using transcriptome sequencing (top) and a Sanger sequencing chromatogram of the fusion junction (bottom).

A

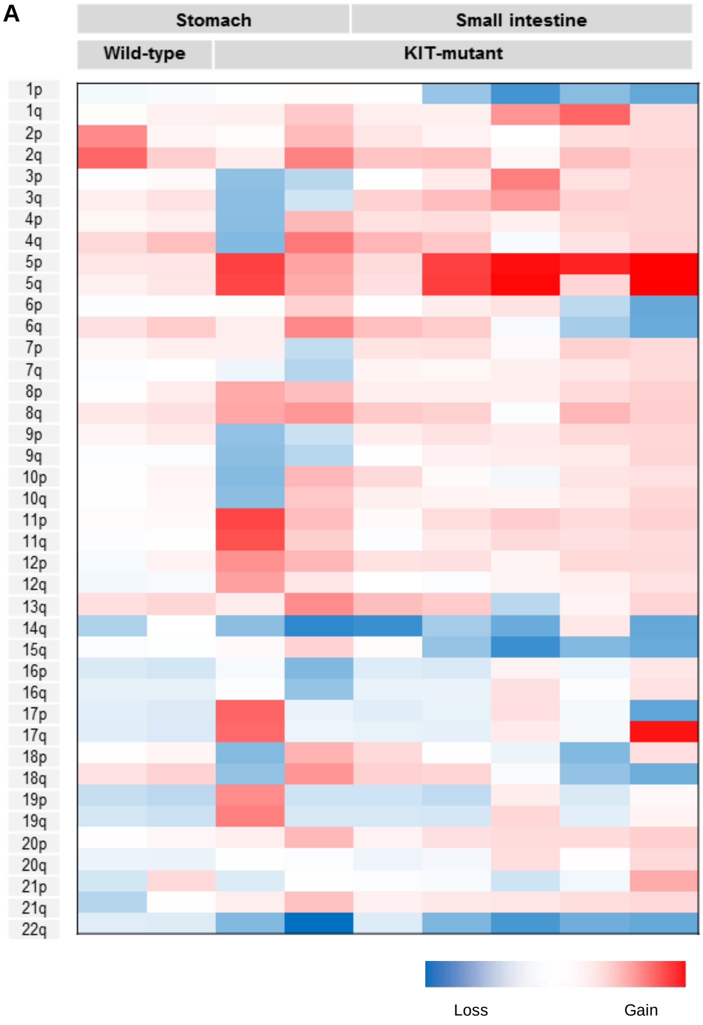

B

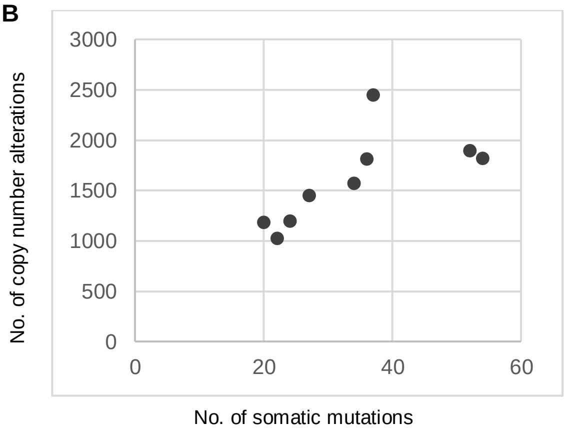

Figure S2: DNA copy number changes in GISTs. **(A)** The heat map shows the alterations in each tumor (horizontal axis) plotted by chromosomal location (vertical axis). **(B)** There is an approximate correlation between the numbers of somatic mutations and copy number alterations.

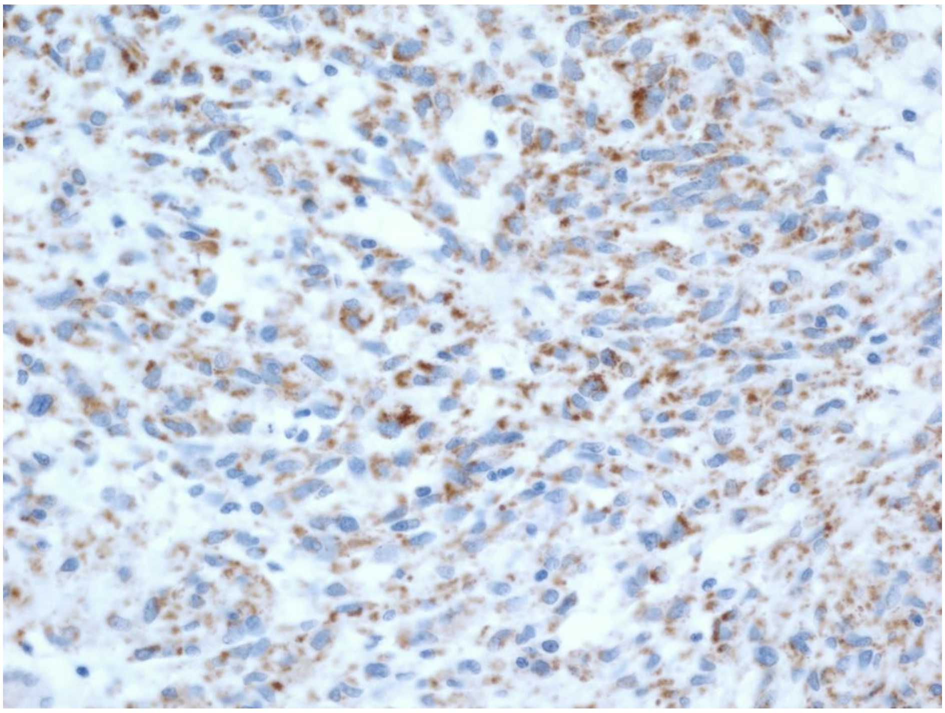

Figure S3: Representative immunostaining result for AMACR. Sample No. 9 shows a distinct granular cytoplasmic staining pattern in tumor cells.

A

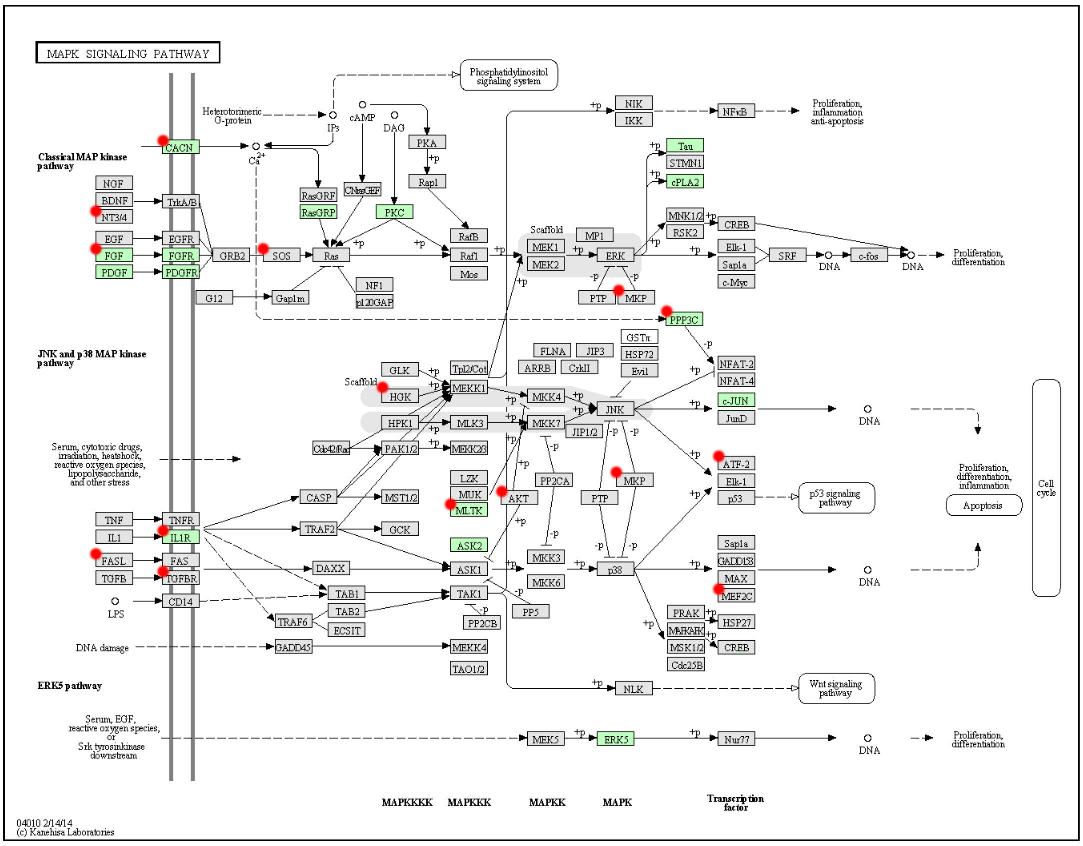

B

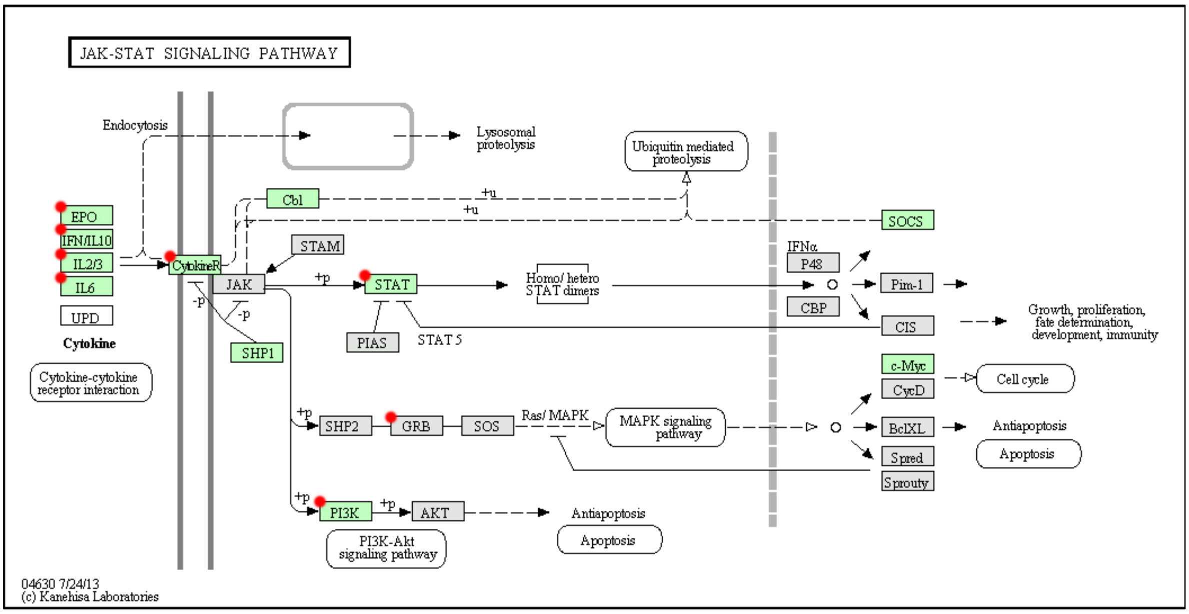

Figure S4: Genes involved in MAPK and JAK-STAT signaling cascades, provided by the KEGG pathway database. The genes overexpressed in gastric wild-type vs. small intestinal *KIT*-mutant samples **(A)** and those overexpressed in small intestinal vs. gastric tumor samples **(B)** are shown in green. The genes with copy number gains are labeled with red dots.

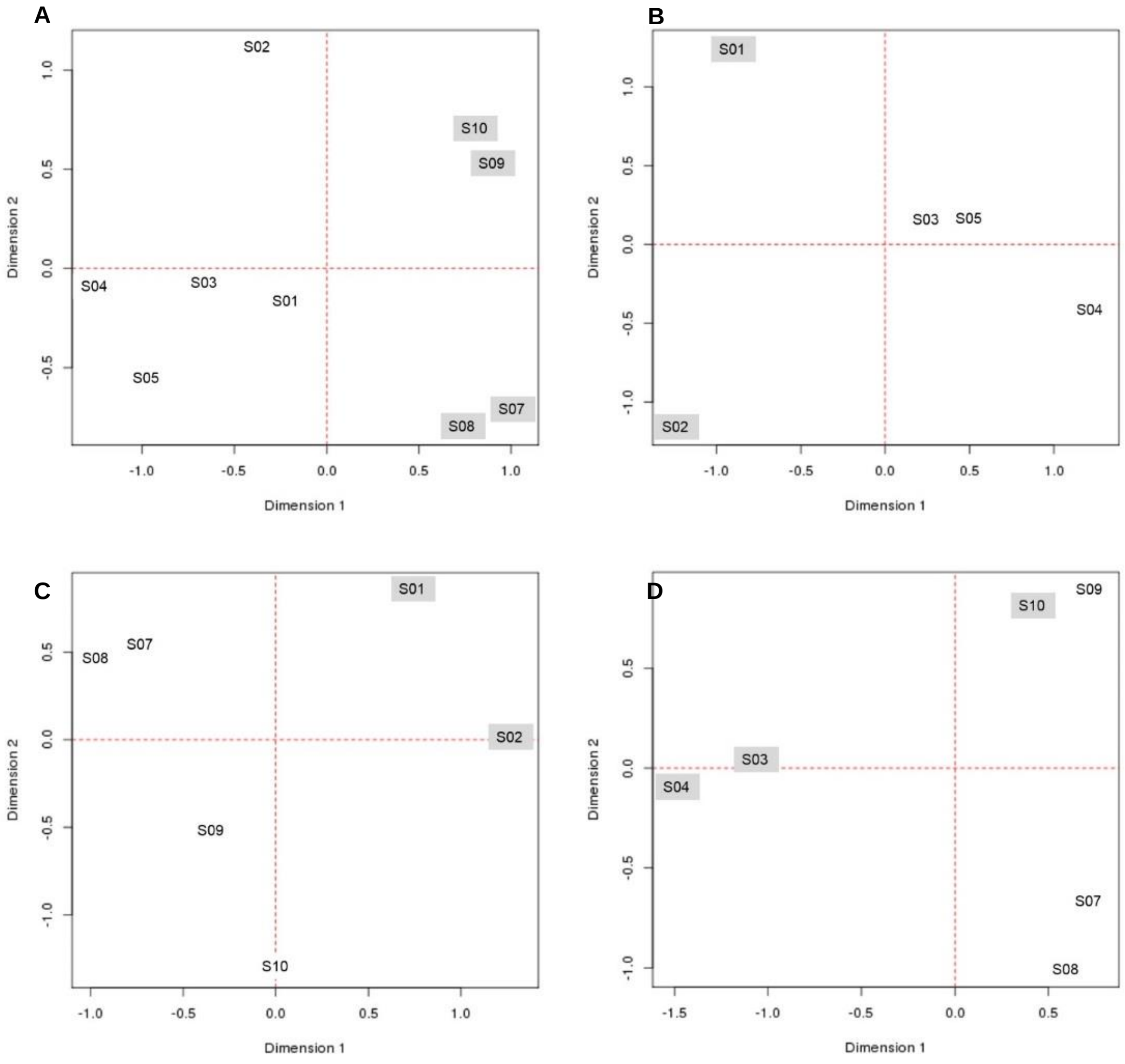

Figure S5: Multidimensional scaling plots of transcriptome expression profiles showing the degree of separation according to tumor location **(A,** stomach vs. small intestine**)**, genotype **(B and C,** wild-type vs. *KIT*-mutant**)**, and imatinib-sensitivity **(D,** resistant vs. sensitive**)**.

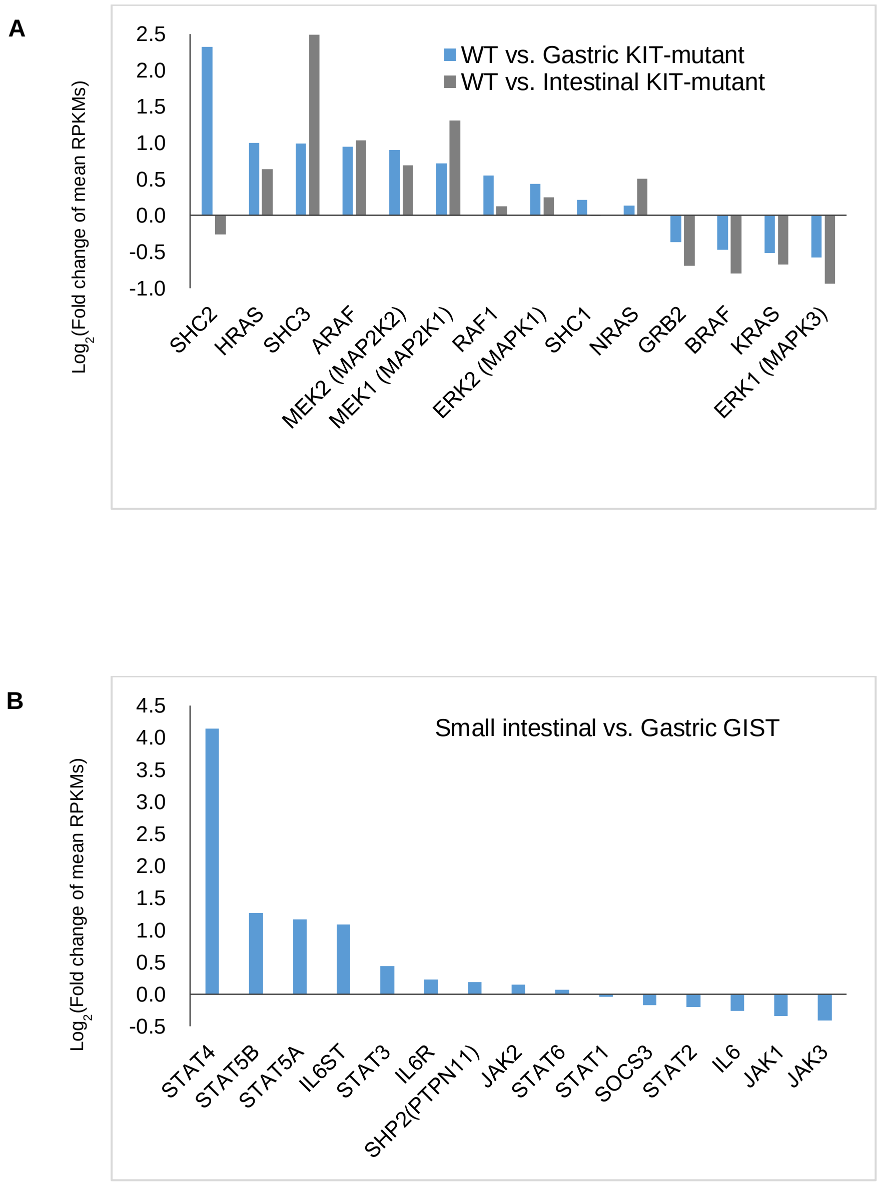

Figure S6: Overexpression of the genes in the MAPK **(A)** and the JAK-STAT **(B)** pathway in wild-type (WT) and small intestinal GISTs, respectively.

Table S1: List of genes with protein-altering somatic mutations in at least two samples.

Gene symbol	Samples mutated	Mutation (amino acid change)	Gene ranker score*	Functional prediction by SIFT	Gene expression (RPKM)
AK2	07	H191D	2.000	DELETERIOUS	91.34
	08	H191D		DELETERIOUS	78.09
	08	K181N		DELETERIOUS	78.09
ALMS1	07	P1968L	not in candidate list	TOLERATED	27.67
	08	P1968L		TOLERATED	53.58
BOC	07	N283K	not in candidate list	TOLERATED	61.45
	08	N283K		TOLERATED	74.90
BTBD3	07	G512V	not in candidate list	DELETERIOUS	826.71
	08	G512V		DELETERIOUS	1260.30
CISD2	07	H114Y	not in candidate list	DELETERIOUS	34.00
	08	H114Y		DELETERIOUS	28.88
DROSHA	07	L706F	0.500	DELETERIOUS	52.18
	08	L706F		DELETERIOUS	82.59
DUX4L2	02	F241L	not in candidate list	TOLERATED	4.26
	10	G231R		TOLERATED	0.73
GPR180	07	S314C	1.000	TOLERATED	26.46
	08	S314C		TOLERATED	20.55
HLA-DQB1	04	P88L	not in candidate list	.	6.85
	10	V235I		.	87.97
IGSF3	07	S651I	not in candidate list	TOLERATED	8.07
	08	S651I		TOLERATED	21.18
LILRA6	07	R205Q	not in candidate list	TOLERATED	3.85
	08	T187N		TOLERATED	0.97
LONRF1	07	K678E	not in candidate list	TOLERATED	39.40
	08	K678E		TOLERATED	43.08
ME3	07	L46Q	1.250	TOLERATED	32.21
	08	L46Q		TOLERATED	44.05
MLL3	03	R909K	4.000	TOLERATED	91.31
	10	N729D		TOLERATED	121.20
OR10S1	07	R305Q	not in candidate list	DELETERIOUS	0.01
	08	R305Q		DELETERIOUS	0.01
PCM1	07	R263T	3.000	TOLERATED	248.74
	08	R263T		TOLERATED	232.17

RAB39B	07	A154D	not in candidate list	DELETERIOUS	14.92
	08	A154D		DELETERIOUS	11.35
REG3A	04	H50P	not in candidate list	DELETERIOUS	0.01
	08	H50P		DELETERIOUS	0.01
RHPN2	07	Q378*	1.000	.	73.63
	08	Q378*		.	72.25
RP1	07	K693N	not in candidate list	DELETERIOUS	0.03
	08	K693N		DELETERIOUS	0.01
	10	S1575F		DELETERIOUS	0.02
RXFP2	07	K385*	not in candidate list	.	0.06
	08	K385*		.	0.04
SETBP1	07	L352M	not in candidate list	TOLERATED	28.77
	08	L352M		TOLERATED	41.95
SGK2	07	R336L	1.000	DELETERIOUS	0.87
	08	R336L		DELETERIOUS	0.41
SHANK2	07	R1244*	0.500	.	94.02
	08	R1244*		.	206.10
SLC35A4	07	V256M	not in candidate list	TOLERATED	76.63
	08	V256M		TOLERATED	146.16
TROAP	07	G374V	1.000	TOLERATED	1.71
	08	G374V		TOLERATED	1.86
WWP1	07	T761K	1.000	DELETERIOUS	57.67
	08	T761K		DELETERIOUS	40.44
ZNF616	07	S419N	1.000	TOLERATED	7.23
	08	S419N		TOLERATED	9.25
ZNF83	01	A277V	1.000	TOLERATED	80.62
	03	R281S		DELETERIOUS	55.82

*http://cbio.mskcc.org/tcga-generanker/

Table S2: Chromosomal distribution of fusion partner genes in nine GIST samples.

**Chromosome**	**No. of genes**																						
	**1**	**2**	**3**	**4**	**5**	**6**	**7**	**8**	**9**	**10**	**11**	**12**	**13**	**14**	**15**	**16**	**17**	**18**	**19**	**20**	**21**	**22**	**X**
All fusions	43	26	23	16	30	22	19	12	32	24	31	29	4	6	17	20	25	4	33	11	6	4	15
Subtype-specific fusions																							
Gastric wild-type	2	2	0	5	1	4	2	1	0	5	4	4	0	2	6	5	5	0	4	2	1	2	2
Gastric KIT-mutant	15	9	6	9	14	15	9	6	10	5	11	7	3	1	8	9	12	2	10	4	3	1	6
Intestinal KIT-mutant	11	7	13	1	9	5	3	3	11	12	12	10	0	1	3	1	6	0	9	1	1	1	5

Table S3:1. Summary of whole-genome sequencing metrics.

Sample	Total reads	Reads aligned (%)	Reads aligned in pairs (%)	Singletons (%)	Median insert size	Bases aligned	Coverage
01_Tumor	573,740,659	98.50%	97.93%	0.57%	323	56,895,697,573	18.38
01_Normal	1,029,081,280	98.85%	98.17%	0.68%	318	102,385,144,264	33.07
02_Tumor	906,958,755	98.70%	97.88%	0.82%	346	90,136,898,989	29.12
02_Normal	926,977,351	98.97%	98.38%	0.59%	341	92,352,212,286	29.83

*The calculation was performed after duplication removal.

Table S3:2. Summary of whole-exome sequencing metrics.

Sample	Total reads	Reads aligned (%)	Bases aligned	On target bases (%)	Mean target coverage	% Target bases >= 2× coverage	% Target bases >= 10× coverage	% Target bases >= 20× coverage	% Target bases >= 30× coverage
01_Tumor	56,122,350	89.37%	5,041,910,165	65.27%	65.61	95.89%	89.64%	81.99%	72.81%
01_Normal	59,231,542	89.44%	5,320,967,469	64.86%	68.76	96.10%	90.29%	83.45%	75.22%
02_Tumor	46,860,061	89.35%	4,207,864,310	65.07%	54.68	95.54%	88.25%	78.66%	66.76%
02_Normal	51,501,829	89.18%	4,616,459,373	64.96%	59.80	95.96%	89.74%	81.65%	71.39%
03_Tumor	56,833,420	89.17%	5,093,434,201	66.08%	67.06	95.95%	89.17%	80.37%	70.27%
03_Normal	48,746,194	88.70%	4,344,977,726	65.69%	56.91	95.76%	88.48%	78.77%	67.18%
04_Tumor	55,375,738	88.91%	4,943,229,943	65.78%	64.84	95.80%	88.75%	79.84%	69.73%
04_Normal	53,121,024	88.48%	4,722,134,538	66.45%	62.50	96.00%	89.56%	81.14%	70.95%
05_Tumor	48,554,293	88.67%	4,326,240,713	66.44%	57.32	95.77%	88.43%	78.60%	66.97%
05_Normal	43,006,121	88.48%	3,819,815,388	65.83%	50.21	95.31%	86.64%	74.81%	61.22%
07_Tumor	63,324,721	90.07%	5,730,366,449	65.19%	74.71	95.40%	88.64%	80.80%	72.23%
07_Normal	63,281,798	89.64%	5,699,169,797	65.09%	74.12	95.58%	89.25%	82.10%	74.14%
08_Tumor	66,199,627	89.62%	5,954,906,125	67.41%	80.44	94.98%	88.23%	80.87%	72.92%
08_Normal	63,281,829	89.64%	5,699,173,867	65.09%	74.13	95.58%	89.25%	82.10%	74.14%
09_Tumor	51,719,990	90.29%	4,692,133,277	67.92%	63.85	95.06%	88.01%	79.42%	69.40%
09_Normal	62,491,263	90.43%	5,679,238,399	67.28%	76.38	95.56%	89.72%	83.33%	76.10%
10_Tumor	58,260,355	88.42%	5,171,420,862	65.72%	67.67	96.08%	89.70%	81.30%	71.50%
10_Normal	49,577,920	88.47%	4,407,317,275	64.81%	56.92	95.87%	88.65%	79.13%	67.64%

*The calculation was performed after duplication removal.

Table S3:3. Summary of whole-transcriptome sequencing metrics.

Sample	Total alignment			Unique alignment				
	Total reads	Reads aligned	Reads aligned in pairs	Unique reads aligned	Reads aligned to '+' strand	Reads aligned to '-' strand	Non-split reads	Split reads
01	81,471,710	69,312,652	61,773,706	67,440,057	33,819,774	33,620,283	53,719,904	13,720,153
02	91,748,166	80,803,907	73,359,660	78,690,856	39,404,509	39,286,347	59,664,503	19,026,353
03	67,358,032	57,004,650	50,454,520	56,179,812	28,116,041	28,063,771	39,772,841	16,406,971
04	78,147,080	68,099,346	61,267,858	66,577,520	33,390,451	33,187,069	49,366,373	17,211,147
05	73,619,860	63,994,753	57,685,638	62,745,608	31,412,390	31,333,218	47,181,717	15,563,891
07	75,354,303	66,906,570	61,151,120	65,317,744	32,678,478	32,639,266	49,732,020	15,585,724
08	79,808,185	71,114,659	64,964,814	69,162,765	34,587,357	34,575,408	52,380,450	16,782,315
09	72,333,630	63,203,359	57,346,574	61,838,806	30,939,640	30,899,166	42,226,203	19,612,603
10	61,279,476	53,989,622	48,961,688	52,781,637	26,398,717	26,382,920	39,542,173	13,239,464
